# Polygamy and Polyandry

**Published:** 1917-01

**Authors:** 


**Polygamy and Polyandry.** Oscar J. Smith, Medico-Legal Jour. states that the former has existed in almost every country and the latter to a considerable extent and persists in the Leutian Islands, Thibet, Ceylon, parts of Africa and some of the South Sea Islands. The former produces the maximum, the latter the minimum birth rate so that the two customs tend to correspond to maximum and minimum food supplies but are also related to the proportionate birth rates of the sexes. In polygamic Morocco, 3 females are born to 1 male, in Thibet, 3 males to 1 female.

